# A systematic review of effective parent-adolescent sexual and reproductive health information communication in lower- and middle-income countries

**DOI:** 10.4102/hsag.v28i0.2435

**Published:** 2023-09-29

**Authors:** Frank B. Agyei, Doreen K. Kaura

**Affiliations:** 1 Department of Nursing and Midwifery, Faculty of Medicine and Health Sciences, Stellenbosch University, Cape Town, South Africa

**Keywords:** Intervention, skills, motivation, effectiveness, teenage, lower-and-middle-income

## Abstract

**Background:**

Parents play an important role in the sexual and reproductive health (SRH) of their adolescents. Parent–adolescent SRH information communication is cardinal and is expected to improve SRH outcomes of adolescents.

**Aim:**

The aim of this systematic review was to search for effective SRH information communication interventions in lower- and middle-income countries (LMICs) to inform the adaptation of parent–adolescent SRH information communication intervention.

**Method:**

This is the first phase of an explanatory sequential mixed method study. The systematic review was carried out by employing Joanna Briggs Institute software for reviews. Search sources included Cochrane Reviews Library, EMBASE, CINAHL, PubMed, OVID, Scopus, Web of Science and Science Direct. A systematic search strategy was formulated, making use of the key terms: adolescent, teenager, youth, parent, mother, father, caregiver, reproductive, sexual, health, information, communication and intervention.

**Results:**

Five articles met the inclusion criteria for full-text screening. The interventions included addressed sociodemographic covariates; parent–adolescent general communication; parental monitoring; parent–adolescent communication about sex-related topics; parent’s sexual communication skills; parent’s self-efficacy in sexual communication; parent’s responsiveness to sexual communication; communication frequency; quality of sex‑related communication and information-motivational-behavioural skills.

**Conclusion:**

Findings suggest that evidence-based SRH information communication interventions are effective in improving parent–adolescent SRH information communication to optimise safe SRH behaviour in LMICs.

**Contribution:**

This systematic review identified effective SRH information communication interventions in LMICs, which can form the basis of further qualitative exploration for adaptation of a culturally sensitive intervention in Ghana.

## Introduction

Adolescence is a period of transition between childhood and adulthood, which occurs between 10 and 19 years of age (World Health Organization 2017:2). Adolescents comprise one-sixth of the world’s population and are considered a great resource for all societies. However, at this stage, they engage in risky sexual practices that expose them to poor sexual and reproductive health (SRH) outcomes (Seif, Kohi & Moshiro 2019:2), especially when there are no support systems that commensurate with their needs.

Most sexually active adolescents in lower- and middle-income countries (LMICs) experience poor SRH outcomes, which include sexually transmitted infections (STIs) including HIV/AIDS, unsafe abortions, and early and unwanted pregnancies (Feroz et al. 2021:2). Also, adolescents are not well informed on SRH issues, with their major source of information being their peers (Kusheta et al. 2019:2). The information they receive from their peers may be inaccurate.

Most adolescents do not seek SRH information before they become sexually active. Their choice of sources of information may depend on the perceived knowledge of sex and confidentiality of the source (Muhwezi et al. 2015:2). Parents as a source of information on SRH for adolescents remain underutilised in LMICs, considering how sensitive a subject it is and the sociocultural issues that surround such discussions (Zakaria et al. 2019:2). There is a perception that the information on SRH will lead to early initiation of sex and encourage adolescents to be promiscuous (Baku, Agbemafle & Adanu 2017:10; Mekonen et al. 2018:5).

Parent–adolescent SRH information communication has gained attention currently and has been found to be one of the strategies that may improve the SRH of adolescents (Kusheta et al. 2019:1). This SRH information communication between adolescents and their parents or guardians has been described as important (Othman et al. 2020:314). Globally, there is agreement about the value of improved parent–adolescent SRH information communication to advance healthy sexual behaviours among adolescents. Adolescents who can communicate with their parents on SRH matters are more likely to avoid the consequences of early initiation of sexual practices (Muhwezi et al. 2015:2). For instance, in Europe and Asia, there is evidence that interventions that trained parents and adolescents in how to communicate SRH information improved their information communication skills and enabled them to communicate SRH information comfortably (Baku et al. 2017:12; Dilorio et al. 2007:1086; Phetla et al. 2008:504; Schuster et al. 2008:6).

The need for interventions to harness and promote parent–adolescent SRH information communication has been highlighted, because of identified low levels of such forms of communication in LMICs (Bogart et al. 2013:7; Coetzee et al. 2014:315). A systematic review conducted in humanitarian and LMIC settings described and evaluated SRH interventions for young people to understand the SRH and psychosocial components of interventions that are effective for improving SRH outcomes (Desrosiers et al. 2020:1–21). The study indicated that several evidence-based SRH interventions may be effective for young people in LMICs. Another systematic review on interventions and strategies to improve SRH outcomes among adolescents living in LMICs also revealed the effect of school and community-based interventions across areas of adolescent SRH rights (Meherali et al. 2021:363–390). It is worth observing that these two studies focused not only on adolescents but also included some groups of older people; their needs may be different. Also, the focus was not on parent–adolescent SRH information communication. This creates a gap that makes this study relevant.

The abovementioned articles highlight that parent–adolescent SRH information communication interventions may be beneficial to reduce adolescent risk behaviours but they should be tailored to the cultural context. It is very important for global health to improve SRH outcomes among adolescents in LMICs. The systematic review therefore aimed to identify effective interventions to improve parent–adolescent SRH information communication to optimise safe and healthy SRH behaviour in LMICs.

Findings from this review can help inform the adaptation of a culturally sensitive parent–adolescent SRH information communication intervention, to enhance parent–adolescent communication in Ghana, which is a LMIC. This is necessary because in Ghana, parents who attempt to discuss SRH issues with their adolescents as well as adolescents who also attempt to discuss issues regarding sexuality with their parents have been described as feeling uncomfortable or awkward and need to be effectively equipped to handle such sensitive issues (Baku et al. 2018:10).

## Methods

The Preferred Reporting Items for Systematic Reviews and Meta-Analyses (PRISMA) and the JBI manual guided the conduct of this systematic review. The review was prospectively registered in the PROSPERO database with registration number CRD42022297526.

### Information sources and search strategy

The search strategy aimed to locate both published and unpublished studies. The researcher developed a full search strategy for Cochrane Reviews Library, EMBASE, CINAHL, PubMed, OVID, Scopus, Web of Science and Science Direct. The search strategy, including all identified keywords and index terms, was adapted for each included database and/or information source. This included (‘Adolescents’ OR ‘Teenagers’ OR ‘Young Adults’) AND (‘Parents’ OR ‘Caregiver’ OR ‘Mother’ OR ‘Father’ OR ‘Guardian’) AND (‘Sexual’ AND ‘Reproductive’ AND ‘Health’) AND (‘Information’ AND ‘Communication’) AND (‘Interventions’ OR ‘Strategies’ OR ‘Best Practices’). The reference list of all included sources of evidence was screened for additional studies. Only studies published in the English language, and only those published between January 2011 and December 2021, were included, as many studies were produced in that period.

### Study selection

Following the search, all identified citations (1706) were collated and uploaded into Mendeley version 1.19.8, and duplicates were removed. Titles and abstracts were then screened by two independent reviewers, F.B.A. and D.K.K., a doctoral student and a Professor, for assessment against the inclusion criteria for the review. Potentially relevant studies were retrieved in full and their citation details were imported into the JBI System for the Unified Management, Assessment and Review of Information (JBI SUMARI). The full text of selected citations was assessed in detail against the inclusion criteria by F.B.A. and D.K.K. Reasons for the exclusion of articles in full text that did not meet the inclusion criteria were recorded. Any disagreements that arose between the reviewers at each stage of the selection process were resolved through discussion. The results of the search and the study inclusion process were reported in the PRISMA flow diagram (Shamseer et al. 2015:9).

### Inclusion and exclusion criteria

Empirical studies included in this review focused on parents and their adolescents regarding SRH information communication interventions in LMICs. Adolescents in this study are defined as persons aged 13 to 16 years. This age group comprises those in the latter stage of early adolescence and in middle adolescence. Because some studies did not specifically include this age group, relevant studies were included if at least 50% of the participants were between the ages of 13 and 16, and if results were stratified according to age groups. Outcomes of the empirical studies were also taken into consideration. This review considered only studies that exposed parents, adolescents (13 to 16 years of age) or both to an SRH information communication intervention, in LMICs. It considered both experimental and quasi-experimental study designs, including randomised controlled trials, in LMICs.

### Assessment of methodological quality and/or critical appraisal

Selected studies were critically appraised by F.B.A. and D.K.K. independently using the Joanna Briggs Institute standardised critical appraisal tools (Munn et al. 2020:2128).

### Data extraction and synthesis

Standardised data such as authors, study aim, participants, and setting and additional information regarding the intervention components and outcomes were extracted from each study. A narrative synthesis approach was used to synthesise data because of diversity in the study outcome and the approach of interventions. Findings were therefore analysed by outcomes measured and components of interventions.

### Ethical considerations

This study was approved by the health research ethics committee of a University in South Africa with reference number, S21/08/159 on 6th January, 2022 and renewal and extension was further granted on 6th January, 2023.

## Results

**FIGURE 1:**
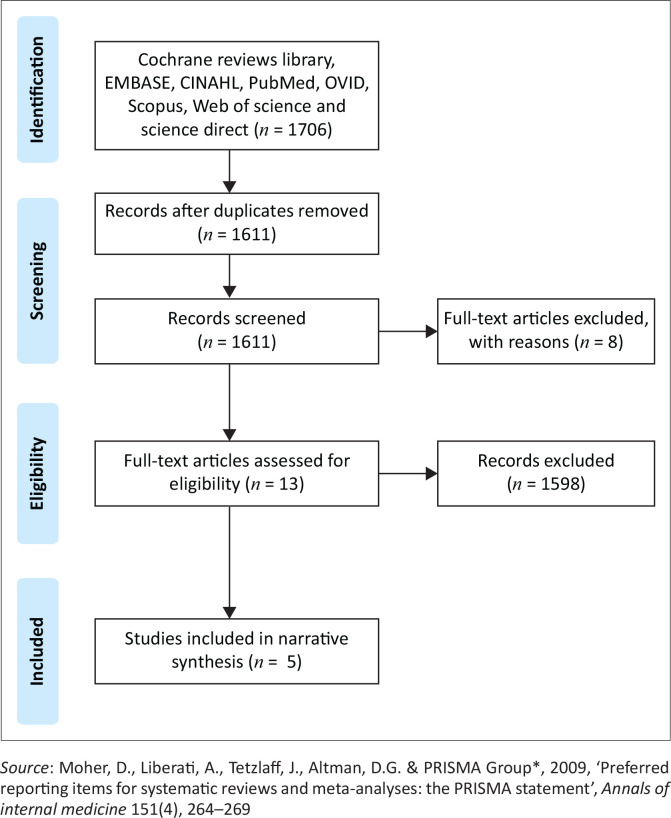
Preferred reporting items for systematic reviews and meta-analyses flowchart for study selection.

Results of the search strategy have been summarised on the PRISMA Flow Chart (see Figure 1). The literature search through all the databases used yielded 1706 results. After the title and abstract screening of the studies, 13 were included. Eight studies were excluded after full-text screening and five were included for the narrative synthesis.

### Study characteristics

The study sought to identify effective interventions to improve parent–adolescent SRH information communication.

Two studies emerged from Iran (Ahari et al. 2020:1–8; Ziaei et al. 2017:1–9), and one each from Tanzania (Seif et al. 2019:1–13), South Africa (Bogart et al. 2013:602–608) and Uganda (Katahoire et al. 2018:91–104), which are all LMICs. Four studies used randomised control trials (see Table 2) and one used quasi-experimental study designs, as shown in table 1. The studies focused on adolescents aged 13–16 (Ahari et al. 2020:1–8), 13 to 15 years (Ziaei et al. 2017:1–9), 12 to 15 years (Katahoire et al. 2018:91–104), 11 to 15 years (Bogart et al. 2013:602–608) and 15 to 19 years (Seif et al. 2019:1–13).

**TABLE 1:** Characteristics of included studies – Quasi-experimental study form.

Study	Country	Setting or context	Participant characteristics	Groups	Outcomes measured	Main description of results
Seif et al. 2019	Tanzania	Unguja-Zanzibar	The study population was all male and female caretakers of adolescents aged 15–19 years	The caretakers were either biological parents or parent figures who had stayed continuously with the adolescents for at least 2 years prior to the survey. Caretakers who were staying with young people who were married were considered ineligible for the study. Participants did not have to be literate to participate. Systematic random sampling was then used to select 28 (1000 participants/36 shehias) households from a sampling frame consisting of approximately 450 houses in each shehia	Information; Motivational; Behavioural skills; SRH communication	No statistically significant finding was observed between the intervention and control groups regarding reporting SRH information (*p* = 0.26), social norms (*p* = 0.51) and perceived efficacy (*p* = 0.33). Motivation post-test (*p* < 0.001) and perceived risk (*p* ≤ 0.001) were however statistically significantly greater in the experimental group compared with the control group, indicating that the intervention group performed better than the control group, although the difference was negligible: a small effect size (*d* = 0.3) was observed. Additionally, a statistically significant finding was observed regarding post-test perceived, behavioural skills, with the intervention group demonstrating greater improvement than the control group (*p* ≤ 0.001) albeit with a small effect size (*d* = 0.2); *F*(1, 827) = 10.81, (*p ≤* 0.001). Post-test SRH communication was statistically significantly greater in the experimental group compared with the control group (*p ≤* 0.01), with a small effect size (*d* = 0.3)

*Source*: Extracted from JBI SUMARI (Joanna Briggs Institute System for the Unified Management, Assessment and Review of Information). Munn, Z., Barker, T.H., Moola, S., Tufanaru, C., Stern, C., McArthur, A. et al., 2020, ‘Methodological quality of case series studies: An introduction to the JBI critical appraisal tool’, *JBI Evidence Synthesis* 18(10), 2127–2133. https://doi.org/10.11124/JBISRIR-D-19-00099

SRH, sexual and reproductive health.

**TABLE 2:** Characteristics of included studies – Randomised controlled trial form.

Study	Country	Setting or context	Participant characteristics	Groups	Outcomes measured	Description of main results
Katahoire et al. 2018	Uganda	The study was conducted in Kampala and Wakiso districts.	Adolescents aged 12–15 years and their parents or caregivers	The study population comprised adolescents in their first year of secondary school, in government-aided day schools, in Kampala and Wakiso districts. A total of 1700 students and parents or caregivers were invited to participate in the study. After random allocation, the intervention group and the control group counted 849 and 851 students, respectively	1. Communication frequency –sex and HIV and/or AIDS related topics 2. Quality of sex‑related communication – Openness, parental competence 3. Positive attitudes towards sex‑related communication 4. Negative attitudes towards sex‑related communication 5. Positive parenting; 6. Parental monitoring 7. Parents’ or caregivers’ legitimacy	Statistically significant findings were observed for both students and parents or caregivers regarding sexuality communication frequency and quality, and for positive and negative attitudes towards sex-related communication. However, the effect sizes were small or negligible, ranging from 0.17 to 0.38
Ziaei et al. 2017	Iran	Gorgan	All mothers in Gorgan covered by health centres and their 13–15-year-old daughters	The participants (336 people in total) of each class were randomly divided into two intervention (84 couples) and control groups (84 couples)	Mother-daughter sex dialogue from the mothers’ viewpoint	One week after the intervention, there was a significant difference in the mean score of mother–daughter sex dialogue between the intervention (34.48 ± 8.74) and control (40.44 ± 9.49) groups (*p* = 0.001) and 1 month after the intervention between the intervention (30.41 ± 10.07) and control (42.47 ± 9.62) groups (*p* < 0.001)
Ahari et al. 2020	Iran	Karaj	Parents of adolescents aged 13–16 years (7th–10th school grade)	The final sample size was estimated at about 43 parents in each group, considering a 20% attrition rate	1. Parent–adolescent general communication 2. Parental monitoring 3. Parent–adolescent communication about sex-related topics 4. Parent’s sexual communication skills 5. Parent’s self-efficacy in sexual communication6. Parent’s responsiveness to sexual communication	In terms of parent–adolescent general communication, parental monitoring, parent–adolescent communication about sex-related topics, parent’s sexual communication skills, parent’s self-efficacy and responsiveness to sexual communication, there were no significant differences between intervention and control groups at the baseline (*p* > 0.05). Compared with controls, intervention parents reported more improvement in general communication across the time; however, significant differences were not observed regarding general communication and parental monitoring (*p* = 0.94, *p* = 0.95). Parents in the intervention group significantly differed from those in the control group for the mean scores of parent–adolescent communication about sex-related topics (*p* = 0.04), parent’s sexual communication skills (*p* = 0.04), parent’s self-efficacy (*p* = 0.002) and responsiveness (*p* < 0.001) to sexual communication at each follow-up
Bogart et al. 2013	South Africa	This study was conducted in Cape Town. City Council worksites in the Western Cape province, which is 27% black African, 54% mixed race and 18% white. Official city languages are English, isiXhosa (spoken by the majority of black Africans in the Western Cape), and Afrikaans (spoken by people who are mixed race). The City is Cape Town’s largest employer, with a workforce of ∼22 000 across multiple locations	Parents of 11–15-year-olds were recruited from five City departments. Employees were eligible if they had one or more children aged 11–15 who resided with them at least 2 days per week. Sixty-six parents (64% male, mean age 43 years [SD = 7], range 23–59) and their 66 adolescents (44% girls; mean age 13 years [SD = 1], range 11–15) participated. They included 34 isiXhosa-speaking and 32 Afrikaans-speaking parent-child dyads; seven parents were nonbiological (four stepparents, three relatives)	Thirty-four parents were randomised to the intervention group. Of those assigned to the intervention group, 68% of parents attended session 1, 76% session 2, 74% session 3, 71% session 4 and 82% session 5. In addition, 73% attended four to five sessions, 15% attended two to three sessions, 3% attended one session and 9% attended no sessions. There were 32 parents in the control group	1. Sociodemographic covariates 2. Communication about HIV and sex 3. Parents’ self-efficacy for condom use 4. Condom use behaviours	Multivariate regressions indicated that the intervention significantly increased parents’ comfort with talking to their adolescent about sex, *b*(SE) = 0.98(0.39), *p* = 0.02 and the number of sex- and HIV-related topics discussed with their adolescent, *b*(SE) = 3.26(1.12), *p* = 0.005. Compared with control parents, intervention parents were more likely to discuss new sex- and HIV-related topics not discussed before the intervention, *b*(SE) = 2.85(0.80), *p* < 0.001. The intervention significantly increased parents’ self-efficacy for condom use, *b*(SE) = 0.60(0.21), *p* = 0.007

*Source*: Munn, Z., Barker, T.H., Moola, S., Tufanaru, C., Stern, C., McArthur, A. et al., 2020, ‘Methodological quality of case series studies: An introduction to the JBI critical appraisal tool’, *JBI Evidence Synthesis* 18(10), 2127–2133. https://doi.org/10.11124/JBISRIR-D-19-00099

SD, standard deviation; SE, standard error.

### Intervention delivery

This section provides information on how the various interventions were delivered to parents and/or adolescents. The discussion will address those who received the interventions, the frequency of sessions, the length and duration of the interventions, and the methods used to deliver the interventions. It also addresses those who delivered the interventions and the settings where the interventions were delivered.

Regarding the participants who received the interventions, three of the studies (Bogart et al. 2013:602–608; Katahoire et al. 2018:91–104; Ziaei et al. 2017:1–9) used both parents or caregivers and their adolescents in the study. Out of these three, Katahoire et al. (2018:91–104) made use of both mothers and fathers and also male and female adolescents. The remaining two studies (Bogart et al. 2013:602–608; Ziaei et al. 2017:1–9) made use of female parents and female adolescents. Two studies (Ahari et al. 2020:1–8; Seif et al. 2019:1–13) made use of only parents or caretakers in the study, who were both males and females.

Regarding the methods of delivery, most interventions used more than one delivery method, including lectures, role plays, group discussions, posters and games. Apart from these delivery methods, in the study by Katahoire et al. (2018:91–104) and Bogart et al. (2013:602–608), take-home assignments were given.

The intervention in the various studies was delivered by experts, including experts in SRH education and adolescent counsellors (Ahari et al. 2020:1–8), a consultant midwifery student with life skills training certification and certification of participation workshops on the sexual training of children and adolescents (Ziaei et al. 2017:1–9), teachers who teach English and Christian Religious Education (Katahoire et al. 2018:91–104) and HIV peer educators (Bogart et al. 2013:602–608). Unfortunately, Seif et al. (2019:1–13) did not report on the persons who delivered the intervention.

Interventions were delivered in a school setting (Katahoire et al. 2018:91–104), worksite (Bogart et al. 2013:602–608), community (Ahari et al. 2020:1–8; Seif et al. 2019:1–13) and health centre (Ziaei et al. 2017:1–9).

### Intervention components

The intervention components are the individual parts or features that influenced the outcomes of the various interventions identified in the review.

The intervention components that were included in the study by Ziaei et al. (2017:1–9) were normal sexual development, relationship between adolescents and parents, communication about sex and sexual risk reduction. Two studies asserted that group counselling should be based on communication skills [integrated SRH issues, discussion between adolescents and their parents about SRH and parenting] (Ahari et al. 2020:1–8; Katahoire et al. 2018:91–104). According to Seif et al. (2019:1–13), SRH information communication is influenced by information, motivation and behavioural skills. In the study of Bogart et al. (2013:602–608), condom use behaviour as an outcome was also influenced by communication about HIV and sex, comfort talking about sex and parent’s self-efficacy for condom use.

In all the identified studies, communication skills stand out in these components as the determinant of information communication, also influenced by factors such as the SRH topics discussed and attitudes towards SRH information communication. These have been developed and expanded in the themes given here.

#### Topics discussed in sexual and reproductive health communication

This refers to the information that was shared between parents and their adolescents. Various topics were discussed across the included studies. In Katahoire et al. (2018:91–104), the following topics were discussed: pregnancy, sex and postponing sex, pregnancy prevention, condom use, sex in exchange for gifts, STIs including HIV and/or AIDS, dating relationships and/or having a boyfriend or girlfriend. In Seif et al. (2019:1–13), the topics were abstinence, safer sex, pregnancy, STIs including HIV, contraceptive usage, abortion and homosexuality. Bogart et al. (2013:602–608) discussed HIV, sex, the changes in adolescence, pregnancy, adolescent decision-making about sex, pregnancy prevention, how condoms prevent HIV, steps in condom use, HIV prevention, what to do if a partner does not want to use a condom, the importance of not pressuring others to have sex, reasons for sex, abstinence, saying no to sex, consequences of alcohol and drug use, recognising violence and abuse in relationships, and homosexuality. Ahari et al. (2020:1–8) did not directly mention all the topics that were discussed; however, it could be deduced that the discussion centred on body changes during adolescence, abstinence and reducing sexual risks. In Ziaei et al. (2017:1–9), participants were taught how to answer girls’ questions concerning matters such as pregnancy, childbirth, delivery of a baby, masturbation, methods of contraception, STIs including HIV and/or AIDS, hepatitis and protection against sexual abuse.

Certain topics were not discussed in some studies, probably because of how sensitive those topics are in relation to the prevailing cultural values of a particular setting.

#### Attitudes towards sex-related communication

One of the communication subthemes of the various interventions was attitudes. This refers to what personally or socially motivates parents and/or their adolescents to communicate. All the studies measured the attitudes of either parents or adolescents towards SRH information communication (Ahari et al. 2020:1–8; Bogart et al. 2013:602–608; Katahoire et al. 2018:91–104; Seif et al. 2019:1–13; Ziaei et al. 2017:1–9).

#### Sexual and reproductive health communication skills

Communication skills are the parents’ and/or adolescents’ objective ability to communicate, and the quality and self-efficacy in communicating. The SRH information communication skills identified included openness and parental or adolescents’ competence in communication (Katahoire et al. 2018:91–104; Ziaei et al. 2017:1–9), self-efficacy (Ahari et al. 2020:1–8; Seif et al. 2019:1–13) and comfort with communication (Bogart et al. 2013:602–608).

The improvement in skills led to an increased frequency of communication, which is one of the indicators of the impact of the intervention.

### Effectiveness of interventions

Effectiveness refers to the degree to which the interventions were successful in maximising parent–adolescent SRH information communication.

The five studies identified reported improvement in outcomes related to SRH information communication. All the studies were randomised control trials, except for Seif et al. (2019:1–13), which used a quasi-experimental approach.

Bogart et al. (2013:602–608) tested whether ‘Let’s Talk’, a worksite-based parenting programme, improves parent–child communication about HIV and sexual health, as well as parent condom use, self-efficacy and behaviour. Regarding the communication on sexual health and HIV, results after the intervention delivery showed that there was a statistically significant difference (*p* = 0.005) in the pre-to-post increases in the intervention means compared with the control means. The number of new topics discussed as baseline was greater among intervention parents (*M* = 5.9, standard deviation [SD] = 4.7) than among the parents in the control group (*M* = 2.8, s.d. = 3.6; *p* < 0.001).

Seif et al. (2019:1–13) assessed the effect of an intervention aiming to improve caretaker-adolescent communication on SRH matters, through improving information, motivation and behavioural skills related to SRH communication. It was found that SRH communication in the experimental group was significantly greater than the control group in statistical terms *F*(1,827) = 16.74; (*p* ≤ 0.01) albeit with a small effect size (*d* = 0.3), which shows that there is a non-overlap of 21.3% in the two distributions. At 6 months, the results showed a statistically significant difference between the experimental and control groups, which favoured the former *F*(1,827) = 17.9; (*p* < 0.001) albeit, also with a small effect size (*d* = 0.3). At 1 year follow-up, the results still demonstrated a statistically significant difference that favoured the experimental group, with a small effect size (*d* = 0.4). The marginal increment in Cohen’s *d* may highlight that the longer the participants receive and practice the intervention, the more they are influenced by it. This may translate to improved outcomes.

Ziaei et al. (2017:1–9) determined the effect of group counselling based on communication skills of mothers, through their sex dialogue with their daughters. It was found that there was a statistically significant difference in the mean score of mother–daughter dialogue on sex one week after the intervention, between the intervention (34.48 ± 8.74) and control (40.44 ± 9.49) groups (*p* = 0.001), and 1 month after the intervention, between the intervention (30.41 ± 10.07) and control (42.47 ± 9.62) groups (*p* < 0.001).

Katahoire et al. (2018:91–104) examined the effect of a school-based intervention aimed at improving aspects of parent or caregiver-adolescent communication on sexuality. Regarding the quality of sex-related communication, in terms of openness and parental competence, the results showed that there were significant effects among parents or caregivers as well as among the students.

The effect sizes were 0.36 (*t* = 5.162; *p* < 0.001) (parents or caregivers) and 0.26 (*t* = 5.279; *p* < 0.001) (students). The study assessed the attitudes towards SRH communication and found that there was a significant difference between the two groups on positive attitudes with effect sizes equal to −0.31 (*t* = 4.424; *p* < 0.001) for parents or caregivers and 0.20 (*t* = 2.772; *p* = 0.006) for students. In the intervention group, the reduction in negative attitudes towards sex-related communication was significantly greater than in the control group, with effect sizes equal to −0.17 (*t* = 2.349; *p* = 0.019) parents or caregivers and −0.19 (*t* = 2.662; *p* = 0.008) for students.

Ahari et al. (2020:1–8) evaluated the effectiveness of a sexuality education programme for parents of male adolescents to promote parent–adolescent sexual communication. It was noticed that the mean scores of parent–adolescent communications about sex-related topics, parent’s sexual communication skills, parent’s self-efficacy for sexual communication and responsiveness to sexual communication increased in at least one of the stages (from the baseline to first and second follow-up) in the intervention group, and that all were statistically significant between the two groups. The researchers conducted a Bonferroni test for pairwise comparisons of the three stages (from the baseline to the first and second follow-up). They found that the mean differences in parent–adolescent communication about sex-related topics and parent self-efficacy for sexual communication proved that parents in the intervention group had greater improvement throughout all three stages (from the baseline to first and second follow-up *p* < 0.001; from the first to second follow-up *p* < 0.05). With regard to parents’ SRH communication skills and their responsiveness, those in the intervention group showed higher mean score changes at the first and second follow-ups than the baseline (*p* < 0.001 for each) but this did not remain statistically significant from the first follow-up to the second (*p* = 0.90 and *p* = 0.31, in the order mentioned).

The effectiveness of these interventions could have been influenced by factors such as the components of the interventions and how the interventions were delivered.

### Summary of findings

**FIGURE 2:**
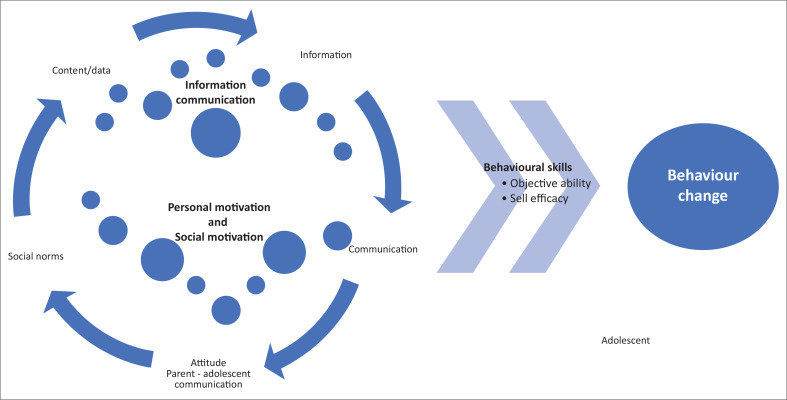
Conceptual framework of sexual and reproductive health information communication skills as deduced from the study.

In summary, the study sought to identify effective interventions to improve parent–adolescent SRH information communication that optimise safe and healthy SRH behaviour in LMICs. The findings highlight that the identified interventions were effective in improving communication. This behavioural change was influenced by the improvement in SRH communication skills, which was evidenced by the improved quality and self-efficacy regarding SRH communication. Sexual and reproductive health communication skill was influenced by the SRH information communicated and participants’ motivation to communicate, expressed as attitudes in most of the studies. This has been presented as a conceptual framework to understand how behavioural change was influenced by the indicated factors (Figure 2).

Regarding the information communicated to recipients of the various interventions, it was also observed that most of the topics were similar, with a few exceptional topics such as alcohol and drug use, sex exchange for gifts, dating and masturbation. It was also significant that Bogart et al. (2013:602–608), Katahoire et al. (2018:91–104) and Ziaei et al. (2017:1–9) reported on studies that included both parents and adolescents. Interventions that educate both parents and adolescents are beneficial; communication should not only be initiated by parents but also by adolescents. Communication will be sustained when both parties have the skill to do so also knowing that, in the end, it will optimise safe and healthy sexual behaviour among adolescents.

## Discussion

The aim of this review was to identify effective interventions to improve parent–adolescent SRH information communication that optimise safe and healthy SRH behaviour in LMICs.

Considering the differing nature of these SRH issues, it may be difficult to draw stronger conclusions regarding the overall effects of the interventions. Additionally, the small effect numbers reported across some studies may highlight a need for cautious interpretation of the review findings. These caveats notwithstanding, the review findings suggest that interventions focused on information communication may be able to improve parent–adolescent SRH information communication.

Most of the studies involved both parents and adolescents in the communication process, and both parents and adolescents were trained. This is effective in promoting communication that can be started by any of the parties. An adolescent may have a need that must be communicated and that cannot wait for the parent to initiate the communication, and vice versa. Involving both parents and adolescents has proven to be effective in the studies identified in this systematic review and other studies (Widman et al. 2016:55). Parent-based studies identified in this systematic review were also effective in optimising communication between them and their adolescents. This also concurs with a study by Santa Maria et al. (2015:39). When parents are well-trained, they can recognise the needs of their adolescents and communicate effectively. This makes them more assertive in supporting their adolescents. Besides, increasing knowledge can improve their self-efficacy in breaking out of the societal code of secrecy surrounding issues of sexuality.

Most of the identified studies used a variety of methods to deliver interventions, such as lectures, role-play and discussions. This correlates with the fact that most intervention studies in this area make use of such delivery methods (Aninanya et al. 2015:3; Mathews et al. 2016:1830). What remains unknown, however, is the impact of each individual mode of delivery on outcomes, considering the sensitive nature of sexuality issues. It was also found in the review that interventions were delivered by teachers, nurses and experts in adolescent health. Generally, intervention studies involving adolescents make use of experts in adolescent health and those who directly care for adolescents. Similar findings were observed in other studies (Ross et al. 2007:1947).

The content, frequency of communication, self-efficacy, attitudes towards sex and communication skills were reported in this study. Most of the identified studies however did not report on what can also be considered non-verbal forms of communication, such as parental monitoring and connectedness. This corresponds to a qualitative review and thematic synthesis by Usonwu, Ahmad and Curtis-Tyler (2021:8) on parent–adolescent communication on SRH in sub-Saharan Africa, which sought to understand the nature and relevance of parent–adolescent SRH communication and the barriers to effective communication. It reported similar forms of communication and intervention components. The reason why parental monitoring and interconnectedness were not included as components is because they have been studied alone as concepts in other studies (Murry et al. 2011:1149; Stanton et al. 2000:18).

The interventions identified in the current review were all effective in training parents and/or adolescents in SRH communication. The findings of this study corroborate the findings of the review conducted by Santa Maria et al. (2015:39) on parent-based adolescent SRH interventions and the effect of communication outcomes. In their review, they found that participants in the intervention group of the studies were 68% more likely than those in the control group to report increased SRH communication, and 75% more likely to report increased comfort. Most interventions in parent–adolescent SRH communication have proven to be effective both in LMICs and advanced countries (Bastien, Kajula & Muhwezi 2011:12–13; Forehand et al. 2007:1125–1127). A variety of SRH topics were discussed in each of the interventions identified in this review. This helps the parents, and their adolescents, to get information on the topics and how to discuss such topics with each other.

The study also shows that the various interventions improved self-efficacy and quality of communication. This was shown in the openness and comfort associated with talking about issues of sexuality. This finding is not different from other study findings. In evaluating a parent-based programme in a similar study, it was noticed that there was parental comfort in communication (Klein et al. 2005:S97). It also corroborates the study by Barr, Johnson Moore and Howard (2012:259–260), which was a pilot project to increase parents’ comfort in communicating with their children about SRH.

The frequency of communication on SRH was another finding of the review. Generally, interventions on sexuality communication increase the rate at which parents and their adolescents communicate on SRH matters (Forehand et al. 2007:1127; McKay et al. 2004:88). This showed a positive attitude towards communication on SRH matters.

The identified interventions that trained parents and/or adolescents were effective in improving parent–adolescent SRH information communication. These interventions can be adapted by countries within the LMICs and be made culturally appropriate for the context. An SRH information communication intervention that is relevant in context may improve SRH outcomes. Therefore, the findings in the study could inform policymaking and further research that can lead to the adaptation of these interventions within a specific culture.

## Limitations

Only studies reported in English were considered; other studies in other languages were therefore excluded. The focus only on quantitative studies could not bring conceptual depth and rich information regarding the feasibility and acceptability of the various interventions. The study was restricted also to interventions in LMICs. The search was limited to randomised controlled trials (with the exception of quasi-experimental studies). The researchers limited the age of adolescents to 13 to 16, which may have excluded some effective SRH interventions for younger or older adolescent populations.

## Conclusion

The review offers a comprehensive summary of effective parent–adolescent SRH information communication interventions in LMICs. It highlights the components of the identified interventions, thematic analysis of the intervention delivery characteristics, frequency, quality, attitudes towards communication on SRH and communication skills. The findings of the systematic review provide information that can guide the adaptation of a culturally sensitive parent–adolescent information communication intervention in the context of Ghana, which is a LMIC and can also inform stakeholders’ decisions to further invest in the adaptation of interventions. This can broaden the understanding of healthcare providers and policymakers about what works most effectively in improving SRH outcomes.
